# Takayasu's arteritis associated with Crohn's disease: a case report

**DOI:** 10.1186/1752-1947-2-87

**Published:** 2008-03-19

**Authors:** Nasser E Daryani, Atoosa Nayer Habibi, Mohammad Bashashati, Mohammad Reza Keramati, Moezedin Javad Rafiee, Hossein Ajdarkosh, Mehrdad Azmi

**Affiliations:** 1 Department of Gastroenterology, Imam Khomeini Hospital, Tehran University of Medical Sciences, Iran; 2 Researcher of Gastroenterology, Imam Khomeini Hospital, Tehran University of Medical Sciences, Iran; 3 Babak Imaging Center, Tehran, Iran; 4 Department of Gastroenterology, Haft-e-Tir Hospital, Iran University of Medical Sciences, Iran; 5 Cancer Institute of Imam Khomeini Hospital, Tehran University of Medical Sciences, Iran

## Abstract

**Introduction:**

The simultaneous presence of Takayasu's arteritis and Crohn's disease in a patient seems to be rare. To our knowledge, no patient with the combination of Crohn's disease and Takayasu's arteritis has been reported from our region.

**Case presentation:**

Herein we present the case of a 22-year-old Iranian woman previously diagnosed as Crohn's disease and who had subsequently developed Takayasu's arteritis.

**Conclusion:**

Clinical suspicion, proper imaging, and consideration of the differential diagnosis are important for the correct diagnosis and management of patients with this coincidence.

## Introduction

Coincidence of Takayasu's arteritis (TA) and Crohn's disease (CD) is exceedingly rare and raises questions about the possibility of similar causes or etiopathogenic mechanisms [1]. The chance of both diseases occurring in the same individual has been estimated to be one in 1010 persons [2]. Recent studies have revealed that 3–9 percent of people with TA may also have CD [2-4]. To our knowledge, there have no reports of simultaneous presence of CD and TA in a patient from our region. Herein we present a case of an Iranian woman previously diagnosed with CD and who subsequently developed TA.

## Case presentation

This patient is a 22-year-old woman with 2 and a half year history of CD who has been treated with sulfasalazine and azathioprine since diagnosis. The diagnosis of CD had been established by colonoscopy which showed regional inflammation and ulceration in the sigmoid colon and the proximal part of the ascending colon. Histology revealed granulomatous colitis, inflamed mucosa of the large intestine with inflammatory epithelial damage, and focal mild crypt distortion. Barium meal and small bowel follow-through were normal.

Since her diagnosis of CD, she has had episodes of relapse with symptoms including fever, weight loss, diarrhoea and arthralgia in the form of inflammatory monoarthritis of the ankle.

Recently the patient presented with symptoms which were different from those seen in her previous relapses. She had malaise, weight loss (7 kg in 6 months), hematochezia and arthralgia, although this time both knees were affected symmetrically with an inflammatory pattern. On physical examination, the patient was afebrile with normal blood pressure. Both radial pulses were normal and symmetrical with a bruit heard over the abdominal aorta, but no carotid or clavicular bruits were detected.

Laboratory tests showed a considerable rise in ESR (100 mm/h) with white blood cell count, 7800/μL; hemoglobin, 10.0 grams/dl; platelets 500000/μL.

Colonoscopy revealed edematous mucosa and ulceration in the ascending colon without any other lesions in the rectosigmoid or other parts of the colon. Barium meal and small bowel follow-through were performed and showed no lesion in the oesophagus, stomach, or small intestine including the terminal ileum and cecum.

**Figure 1:**
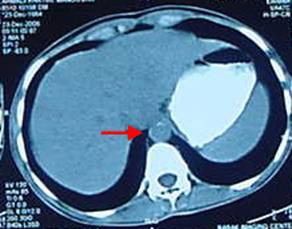
Abdominal CT scan: thickening of the aortic wall (arrow).

Abdominal CT scan showed thickening of the abdominal aorta (4 mm) and narrowing of renal arteries which was suggestive of TA (Figure 1).

**Figure 2:**
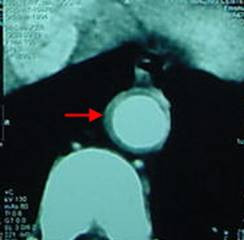
thoracic CT angiography: thickening of the aortic wall (arrow).

To confirm the diagnosis of TA, a CT angiography was also done, which showed marked circumferential mural thickening in branches of the thoracic aortic arch (left common carotid artery and subclavian arteries), right innominate artery, aortic arch and descending aorta which was consistent with Takayasu's arteritis (type v, diffuse involvement) (Figure 2).

With the diagnosis of Takayasu's arteritis, in association with Crohn's disease, oral prednisolone (30 mg/day) was started plus continuation of her previous medication, i.e. sulfasalazine (3 g/day) and azathioprine (100 mg/day). She became symptomless after 4 weeks of treatment, at which time the prednisolone dose was tapered and discontinued over 8 weeks. Her treatment was continued with sulfasalazine and azathioprine with dosage of 3 g and 100 mg per day respectively. Five months after discontinuing prednisolone she has remained symptomless with a normal ESR.

## Discussion

The first reported case of concomitant occurrence of inflammatory bowel disease (IBD) and Takayasu arteritis was in a 35-year-old woman with ulcerative colitis (UC) in 1991 [5]. To our knowledge, 37 patients with UC and TA have been reported since then. In a literature review, 25 patients were reported with concomitant CD and TA. Although, TA rarely occurs in a concomitant manner, this should not be perceived as an unexpected association [3,4].

In our clinic, about 120 patients with CD were registered over a six year period, and the reported case was the first concomitant occurrence of CD and TA.

According to previous studies, Takayasu's arteritis mostly presents simultaneously or after presentation of CD. It is unlikely to precede CD manifestations. It seems that one disease triggers or worsens the other [2]. Unfortunately, there have been no prospective studies to prove this phenomenon.

Most of the reported patients with CD and TA were young women with early manifestations of TA [2,6]. As TA mostly presents in young women [7], it could be suggested that patients with inflammatory bowel diseases would show features of TA in the same age-sex pattern of the IBD.

TA mainly manifests with an absent radial pulse, fever, arthralgia, night sweats and myalgia [7], but in people with Crohn's Disease it may be clinically silent [2]. Due to the involvement of the aorta and its vasculature, TA may present with gastrointestinal bleeding [6]. Since most of these features may also be present in CD, there might be a diagnostic dilemma when there is presentation of both CD and TA.

HLA haplotypes (HLA-A25, B52, DW12, and DR2), and the involvement of immune complexes, and organ specific antibodies have been reported [1,2,8,9]. However, no direct genetic link has been reported and further studies on these associations are recommended [2]. Granulomas and granulomatous vasculitis are seen in both CD and TA. Granulomatous vasculitis shows a common pathophysiology in both diseases [6]. Location of the lesion and sometimes the age of the patient, are two factors that can differentiate between TA and other forms of vasculitis which may coexist with CD, i.e. giant cell (temporal) arteritis is the most difficult vasculitis to distinct from TA but it primarily involves external carotid artery branches and mostly occurs after 50 years of age [10,11]. Moreover, TA can be distinguished from temporal arteritis by predominant lesions at the media-adventitia junction on pathological evaluation [9], although this is mostly impractical.

Corticosteroids are the mainstay of treatment in active TA, and cytotoxic agents are reserved for patients with steroid resistance or who experience relapse [7]. Anti-tumor necrosis factor (TNF) agents might be another treatment possibility [7,12]. Recently, a study which assessed the effect of azathioprine and prednisolone in combination on TA showed that this combination is effective in controlling systemic symptoms and disease activity [13]. Therefore, the drugs that are utilised to treat TA and CD are often the same. Although diagnosis of the diseases in combination may not make any obvious difference in an individual patient's treatment, early diagnosis is critical, because TA can compromise intestinal vasculature and may present with gastrointestinal bleeding, complicating the diagnosis and perhaps exacerbating the clinical course of patients with IBD [6].

## Conclusion

Takayasu's arteritis affects the aorta and its vasculature and may compromise intestinal vessels. Therefore, it may present with gastrointestinal bleeding and exacerbate the clinical course of patients with inflammatory bowel disease. Clinical suspicion, proper imaging, and consideration of the differential diagnosis are important for the correct diagnosis and management of patients with both Takayasu's arteritis and Crohn's disease.

## Competing interests

The author(s) declare that they have no competing interests.

## Authors' contributions

All authors contributed equally to this case report except for MRK who contributed on the revision of the manuscript

## Consent

Written informed consent was obtained from the patient for publication of this Case report and any accompanying images. A copy of the written consent is available for review by the Editor-in-Chief of this journal.
